# Nature Degradable, Flexible, and Transparent Conductive Substrates from Green and Earth-Abundant Materials

**DOI:** 10.1038/s41598-017-04969-y

**Published:** 2017-07-10

**Authors:** Bing Yang, Chunhua Yao, Yanhao Yu, Zhaodong Li, Xudong Wang

**Affiliations:** 1 Department of Materials Science and Engineering, University of Wisconsin-Madison, Madison, WI 53706 USA; 2 School of Power and Mechanical Engineering, Wuhan University, 430072 Wuhan, China

## Abstract

The rapid development of wearable and disposable electronic devices and the rising awareness of environmental sustainability impose growing new demands on the nature degradability of current electronic and energy systems. Here we report a new type of flexible transparent conductive paper completely made from green and earth abundant materials which are also fully degradable and recyclable. Aluminum-doped zinc oxide (AZO) was deposited by low-temperature atomic layer deposition (ALD) as the transparent conductive oxide (TCO) layer on transparent cellulose nanofibril (CNF) papers. The mesoporous structure of the CNF paper rendered strong adhesion of the AZO layer and exhibited excellent mechanical integrity and electrical conductivity within a wide range of tensile and compressive strains. The AZO-CNF paper could be completely dissolved in warm city water after one-hour stirring, demonstrating an excellent nature degradability. A flexible and transparent triboelectric nanogenerator (TENG) was further fabricated using such AZO-CNF papers with a performance that was comparable to other synthetic polymer-based systems. This work illustrated a new and promising strategy of utilizing 100% green and degradable materials in novel electronic and energy harvesting devices.

## Introduction

The rapid evolution of flexible and wearable electronic and optoelectronic devices currently imposes enormous demands on flexible, transparent and conductive substrates as the electrodes for light emitting diodes (LEDs), touch screens, energy harvesters, electronic papers, and artificial skins^1–10^. Transparent flexible electrodes usually include a transparent conductive oxide (TCO) coating on flexible substrates^11^. As a typical TCOs electrode material, indium tin oxide (ITO) has been broadly used in transparent flexible electrodes owing to its superior electrical conductivity and optical transparency^12^. As Indium is a limited natural resource on Earth, alternative transparent conductive coatings have also received intensive research and commercialization interests. Promising examples include fluorine doped Tin Oxide (FTO), aluminum doped zinc oxide (AZO), silver nanowire meshes and graphene^5, 13^. Among them, AZO possesses unique advantages such as the potential to achieve a very low resistivity (on the order of 10^−4^ Ω · cm) and inclusion of inexpensive and earth-abundant non-toxic elements, and thus it received considerable research efforts on the synthesis and quality control as a transparent electrode material^14–20^.

A subcategory in flexible transparent electrodes development deals with the degradability and recyclability, in response to the increasing demands from wearable and disposable electronic devices. Nevertheless, current flexible substrates in electronics mostly use synthetic polymers such as polyethylene terephthalate (PET) and polyethylene naphthalate (PEN) owing to their good resistance to solvents and an excellent tolerance to temperature when coated with TCOs^21^. Degradable flexible transparent substrate is currently underdeveloped. Cellulose, as the most abundant natural polymer, is considered as a promising renewable and green raw material in various industrial applications to address the environmental and recycling issues^22^. Cellulose paper formed by cellulose nanofibrils (CNFs) offers a unique combination of advantages including excellent flexibility, light weight, low cost, and facile recyclability^23^. Recent pioneer developments in electronics and energy-related fields have started to utilize and integrate CNF paper as the substrate in device fabrication, such as flexible transistors^24^, Li-ion batteries^25^, supercapacitors^26^, solar cells and nanogenerators^27^. A recent work further integrated graphene with CNF papers and initially demonstrated the potential of using CNF paper as a transparent electrode^28^. However, despite the tremendous application potential, there is no systematic investigation of developing CNF paper-based flexible transparent conductive substrates. In this paper, we present a development of flexible and nature degradable transparent electrodes based on CNF paper. An AZO thin film was grown by atomic layer deposition (ALD) on a CNF paper as the TCO layer. The AZO-CNF electrode exhibits outstanding optical transmittance (92% at 550 nm) and electrical conductance as well as excellent mechanical integrity within a broad range of tensile and compressive strains. The AZO-CNF paper can be readily dissolved in warm city water showing a good nature degradability. They were further used to fabricate a triboelectric nanogenerator (TENG) device revealing a promising application potential as flexible transparent electrodes in electronic devices.

## Results

**Figure 1:**
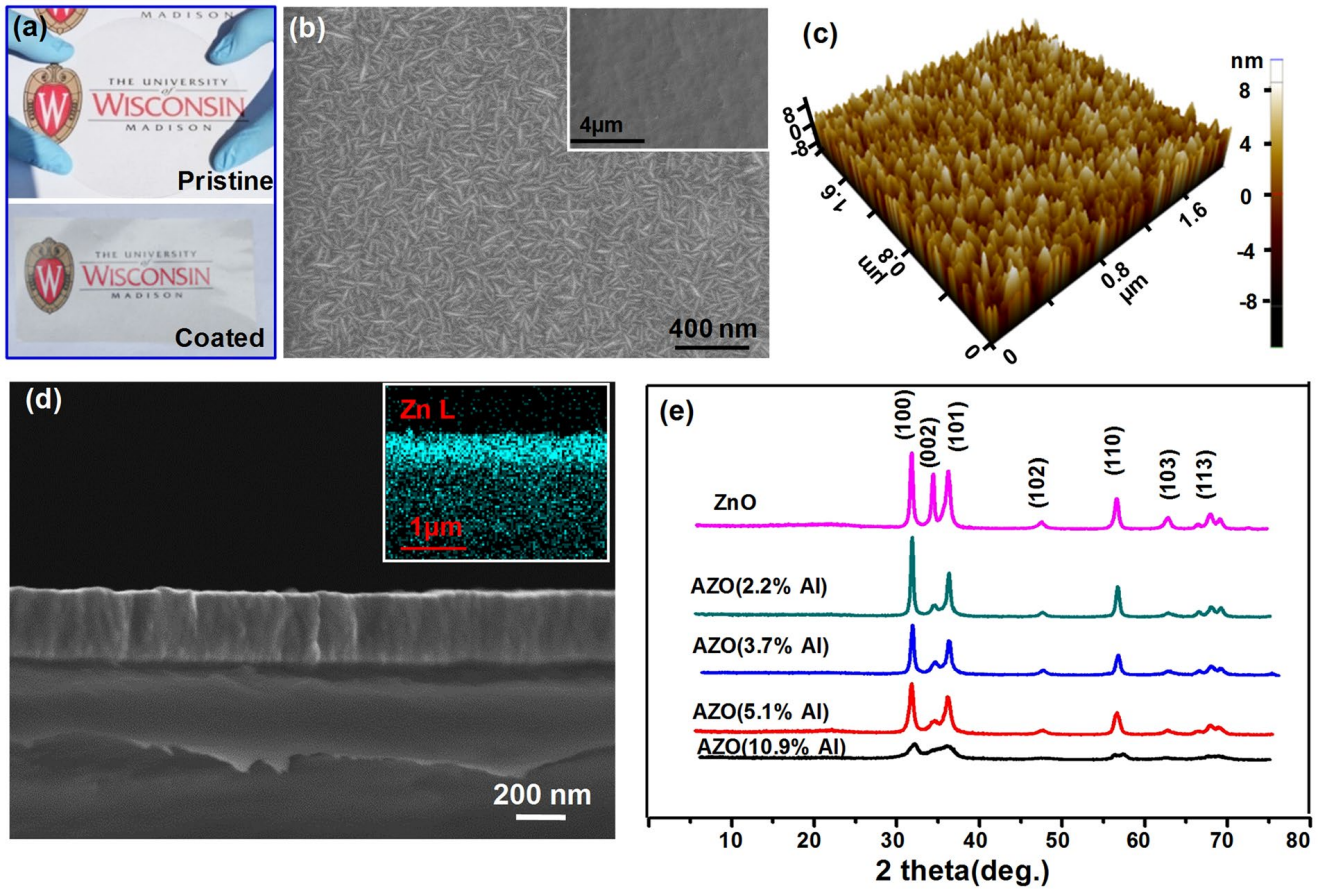
Morphology and crystal structure of AZO-CNF papers. (**a**) Optical image of an as-fabricated CNF paper from the CNF gel (Top) and an AZO-coated CNF paper (Bottom). (**b**) SEM image of the AZO surface with 3.7 at.% Al showing the typical worm-like surface morphology. Inset is a low-magnification SEM image showing the flat surface over a large area. (**c**) AFM image of the AZO surface with 3.7 at.% Al showing uniformly-distributed nanometer-scale grain features with a very low roughness. (**d**) A cross-sectional image of the AZO-CNF paper. The AZO coating exhibited excellent adhesion on the CNF substrate and no crack could be identified. Inset is the corresponding EDS mapping of the Zn elements showing substantial Zn signal within the CNF paper. (**e**) XRD spectra of AZO-CNF papers with different Al concentration.

A 50-µm thick CNF paper with high transparency was used as the supporting substrate for AZO deposition (Fig. 1a, top). A thin layer of AZO with a thickness of 285 nm was deposited by low-temperature ALD (see Method section for synthesis details), which was able to offer excellent film uniformity and conformality^29^. After nearly 27-hour AZO coating at 150 °C, the CNF paper remained a good mechanical integrity as well as a high optical transparency (Fig. 1a, bottom). Low-magnification scanning electron microscopy (SEM) image showed a fairly smooth surface of the as-deposited surface (inset of Fig. 1b). No additives or large variations could be observed. The Al content in the ZnO film was adjusted by alternating the ALD cycle ratio of ZnO to Al_2_O_3_, which was tuned from 20:1,15:1, 10:1 to 5:1. Regardless of the large span of Al composition ratio, the overall film growth rate remained a nearly constant value of ~0.18 nm per cycle, which gave a ~285 nm film thickness on the CNF surface from 1600 total cycles of ALD. However, the Al composition introduced obvious influences on the AZO surface morphology. The un-doped ZnO film exhibited a prolonged prism-like surface feature with a characteristic dimension of ~30 nm × 350 nm, which was typical for low-temperature ZnO ALD thin films (Fig. S1a)^30^. For the AZO film with different Al contents, the surface exhibited a similar worm-like grain feature (Fig. S1b–d). The grain size of AZO with 3.7% Al content were around 50 nm to 150 nm in length and 16 nm to 20 nm in width, which were uniformly distributed across the entire surface (Fig. 1b). When the Al content increased to 10.9 at.%, the length of the grain decreased down to ~60–80 nm, while the width of grain remained the same. The surface morphologies obtained from different Al concentrations were further studied by atomic force microscopy (AFM). For the undoped ZnO film, the surface was covered with randomly-distributed islands giving a root mean roughness (RMS) of ~5 nm (Fig. S2a). Introducing Al content into the film significantly reduced the RMS to ~2 nm with a markedly uniform grain size distribution (Figs 1c and S2b–e). In general, increasing Al concentration reduced the mean grain size and improved the surface flatness, which were desired for achieving high transparency^15^.

The cross-sectional image revealed good adhesion of the AZO film on the CNF surface (Fig. 1d). No crack or peeling was observed at the interface. The grains exhibited a typical columnar structure. At high Al concentrations (>5.1%), the grains became less distinguishable as shown in Fig. S3a–d. From the energy disperse spectrum (EDS) mapping (inset of Fig. 1d), appreciable amount of Zn signal could be detected inside the CNF film area, owing to the mesoporous structure of the CNF paper which facilitated Zn precursor infiltration. Growth of ZnO a certain distance into the CNF paper could enhance the binding strength between the AZO layer and the CNF surface and improve its mechanical integrity.

It was known that Al doping in ZnO could lead to either substitution of Al^3+^ ions for Zn^2+^ ions or interstitial Al defects in the ZnO lattice^31^. To confirm the Al dopant state in the ALD AZO films, crystal structure analysis was conducted by X-Ray diffraction (XRD). A series of XRD spectra obtained from samples with different Al concentrations is shown in Fig. 1e. The hexagonal wurtzite phase of ZnO was confirmed from all deposited samples, where the (100), (002), (101), (102), (110), (103) and (113) peaks were distinguishable. With the increase of Al concentration, the intensity of all diffraction peaks decreased gradually; whereas no obvious peak shifts and additional peaks from crystalline Al_2_O_3_ or ZnAl_2_O_4_ could be observed, suggesting that Al existed as substitutional defects and they reduced the crystallinity of AZO film as the concentration increased^32^.

### Electrical properties of the AZO-CNF

**Figure 2:**
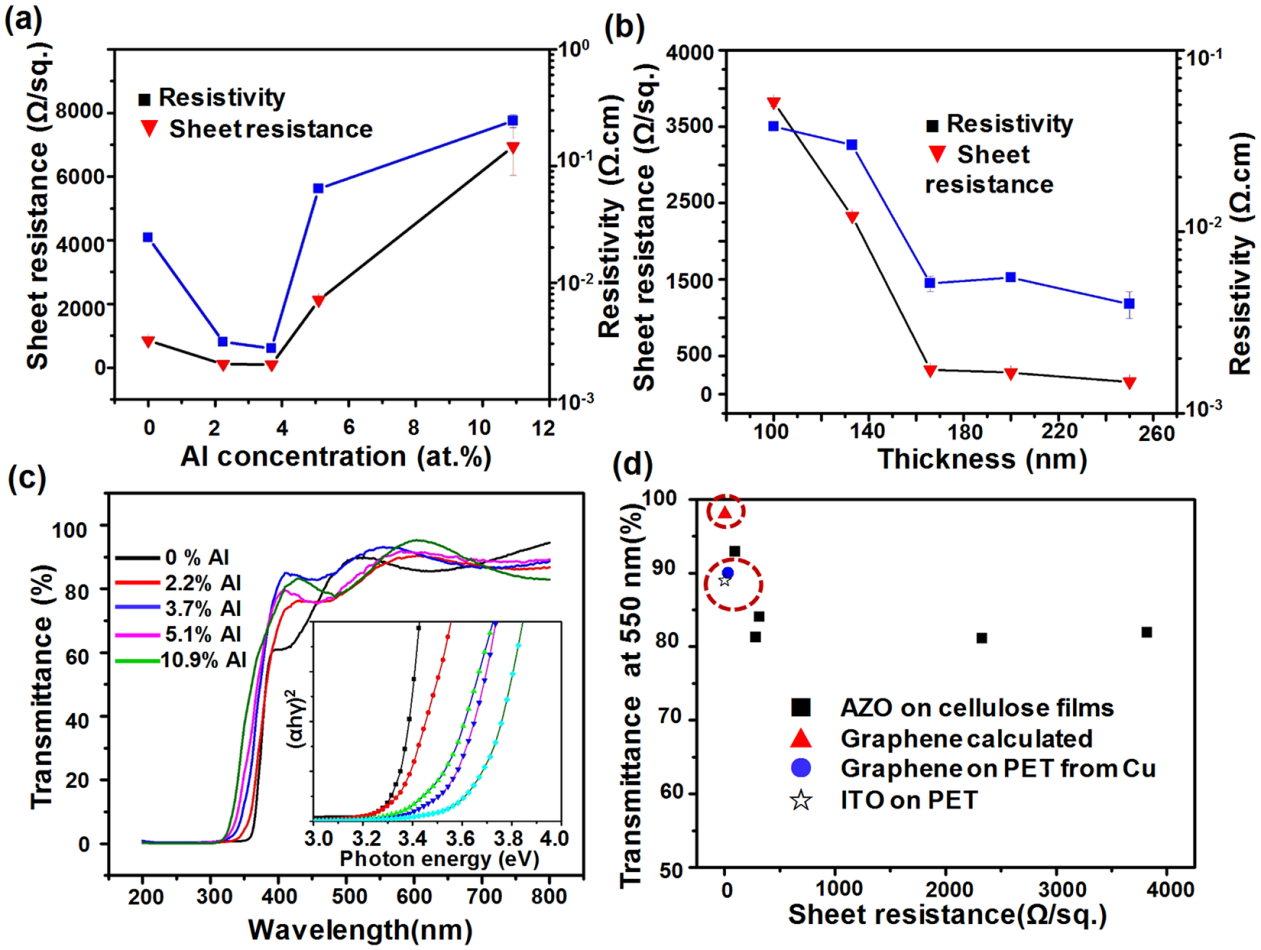
The electrical and optical properties of AZO-CNF papers. (**a**) The sheet resistance and resistivity measured as a function of Al concentration. (**b**) The sheet resistance and resistivity measured as a functional of AZO film thickness. All the AZO films had an Al concentration of 3.7 at.%. (**c**) Transmittance of the AZO-CNF papers with different Al concentrations. Inset is the corresponding Tauc plots revealing the bandgap change. (**d**) Transmittance (at 550 nm) and sheet resistance of AZO-CNF in comparison with other typical flexible transparent electrode candidates.

The electrical resistivity of the AZO-coated CNF papers was investigated by the four-point probe method (see the methods section for details). As shown in Fig. 2a, the sheet resistance (*R*s) and resistivity decreased initially with the addition of Al content and reached the lowest values of *R*s (96 Ω) and resistivity (2.7 × 10^−3^ Ω · cm) at 3.7 at.% Al, suggesting a successful doping of Al atoms in ZnO lattices^33^. Further addition of Al from 3.7 to 10.9 at.% resulted in a sharp increase of *R*s to 6942.6 Ω and resistivity to 2.4 × 10^−1^ Ω · cm. This was likely due to the clustering of AlO_x_ compounds at higher Al concentrations and the reduced crystallinity^34, 35^. By keeping the Al concentration at 3.7%, the film thickness influence was studied. It was found that *R*s decreased sharply when the film thickness increased from 120 nm to 180 nm, and then remained nearly constant as the thickness further increased. A similar variation in sheet resistance as a function of thickness has been reported for ITO films before^36, 37^. This observation was in a good agreement with the Fuchs-Sondheimer theory, which states the main electron scattering mechanism in a polycrystalline film is from the grain boundaries^38, 39^.

Optical transmittances of the AZO-CNF papers were measured at room temperature in the wavelength range from 200 nm to 800 nm. All the samples measured here were grown by the same number of ZnO cycles (1500) with a series of Al concentration from 0% to 10.9%. As shown in Fig. 2c, all the AZO-CNF paper showed a high transmittance of >84% in the visible range of 400–760 nm, which made them acceptable for TCOs applications like displayers and solar cell windows. The fluctuation of the absorption curve could be attributed to the uniform thickness-induced optical interference. Tauc plots were used to reveal the bandgap change as a result of Al doping by assuming that the absorption coefficient *α* ~ −ln T was corresponded to the direct band gap of AZO^40^. A plot of [*α* × (*hʋ*)]^2^ against the photon energy (*hʋ*) was shown in the inset of Fig. 2c. The absorption edge exhibited a blue-shift with increased Al doping concentration, revealing that the bandgap of AZO thin films raised from 3.24 eV to 3.60 eV as the Al concentration increased from 0 to 10.9 at.%. The plots of optical transmittance at 550 nm versus *R*s for different AZO-CNF papers were given in Fig. 2d together with other common transparent conductive substrates including ITO and graphene on PET substrates for comparison^41, 42^. The transmittance of the AZO film with 3.7 at.% Al exhibited the best *R*s and transparency combination, which was at the same level of the reported values of ITO and graphene.

**Figure 3:**
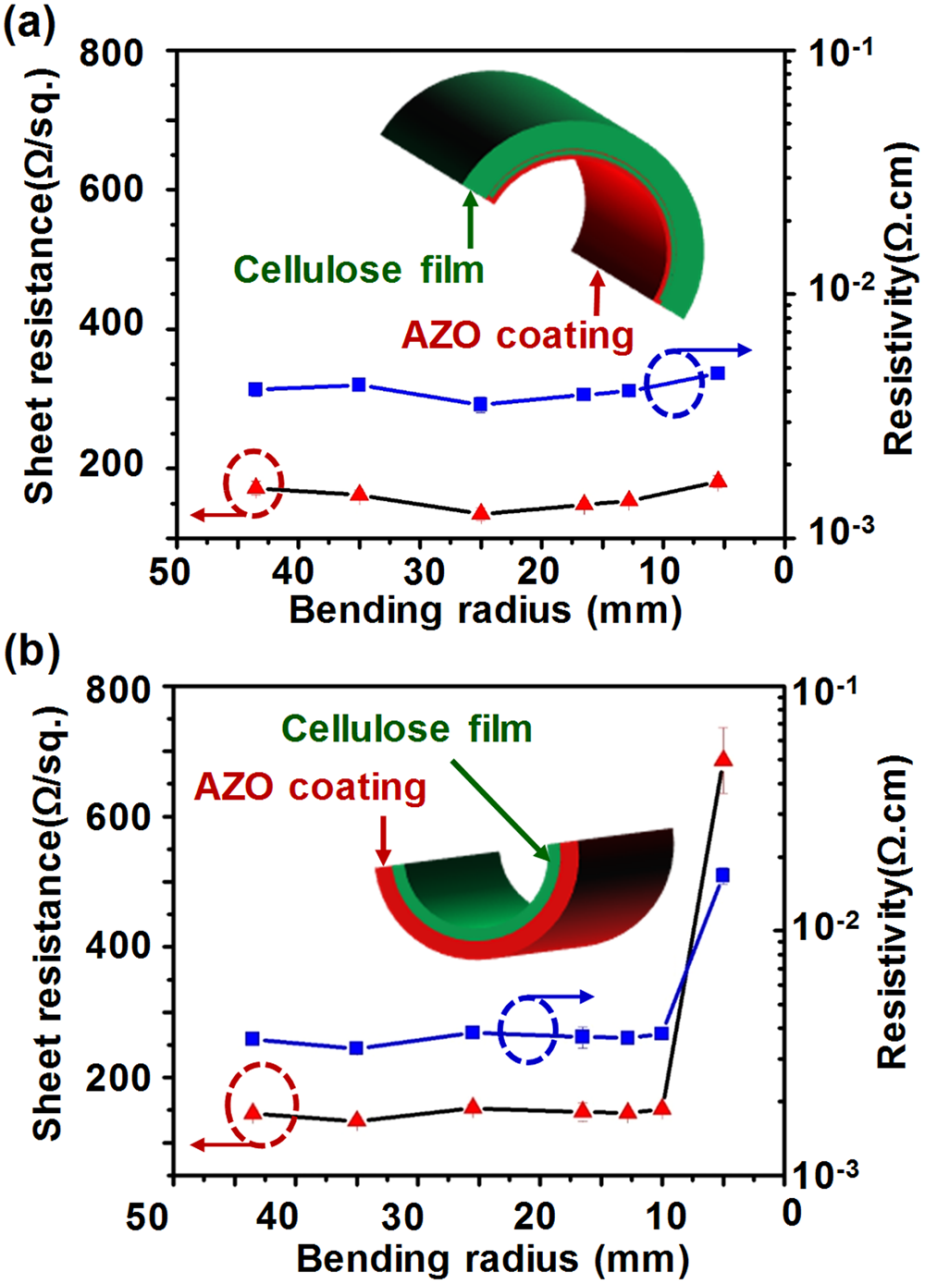
The strain tolerance of AZO-CNF papers at different bending radii. (**a**) Change of Rs and resistivity as a function of bending radii when the AZO film was under compressive strains. (**b**) Change of Rs and resistivity of coating as a function of bending radii when the AZO film was under tensile strains.

Another essential factor of flexible TCO substrates is how the strain influences the conductivity. To determine *R*s as a function of bending radius, a CNF paper coated with a 285 nm AZO (3.76 at.% Al) film was wrapped on cylindrical glass molds with defined radii to test the strain tolerance of the AZO coating. The AZO-CNF paper was wrapped with the AZO side down to introduce compressive strains to the AZO film and with the AZO side up to introduce tensile strains. Corresponding *R*s as a function of bending radius are shown in Fig. 3a (compressive strain) and 3b (tensile strain), respectively. *R*s of the AZO-CNF papers exhibited an excellent stability within the range of 135 to 180 Ω · sq^−1^ under compressive strain when the bending radius was varied from 45 to 5 mm (Fig. 3a). Given the AZO-CNF paper thickness of 50 μm, this radius range corresponded to compressive strains from −0.057% to −0.457% (Fig. S4). Similar stability on *R*s was also observed when the AZO-CNF paper was under tensile strains with the bending radius larger than 10 mm, corresponding to tensile strains less than 0.196% (Fig. 3b). However, *R*s significantly increased as the bending radius further reduced to 5 mm (0.45% tensile strain), indicating degradation of AZO film integrity. The lower tolerance to tensile strain is a typical ceramic behavior where the film quality is dictated by one major defect propagation. Nevertheless, the stable strain region of the AZO-CNF film was comparable to typical ITO-based flexible transparent films^43^, suggesting its good potential to be used as a competitive new type of flexible transparent substrate.

### Degradability and Applications of AZO-CNF papers

**Figure 4:**
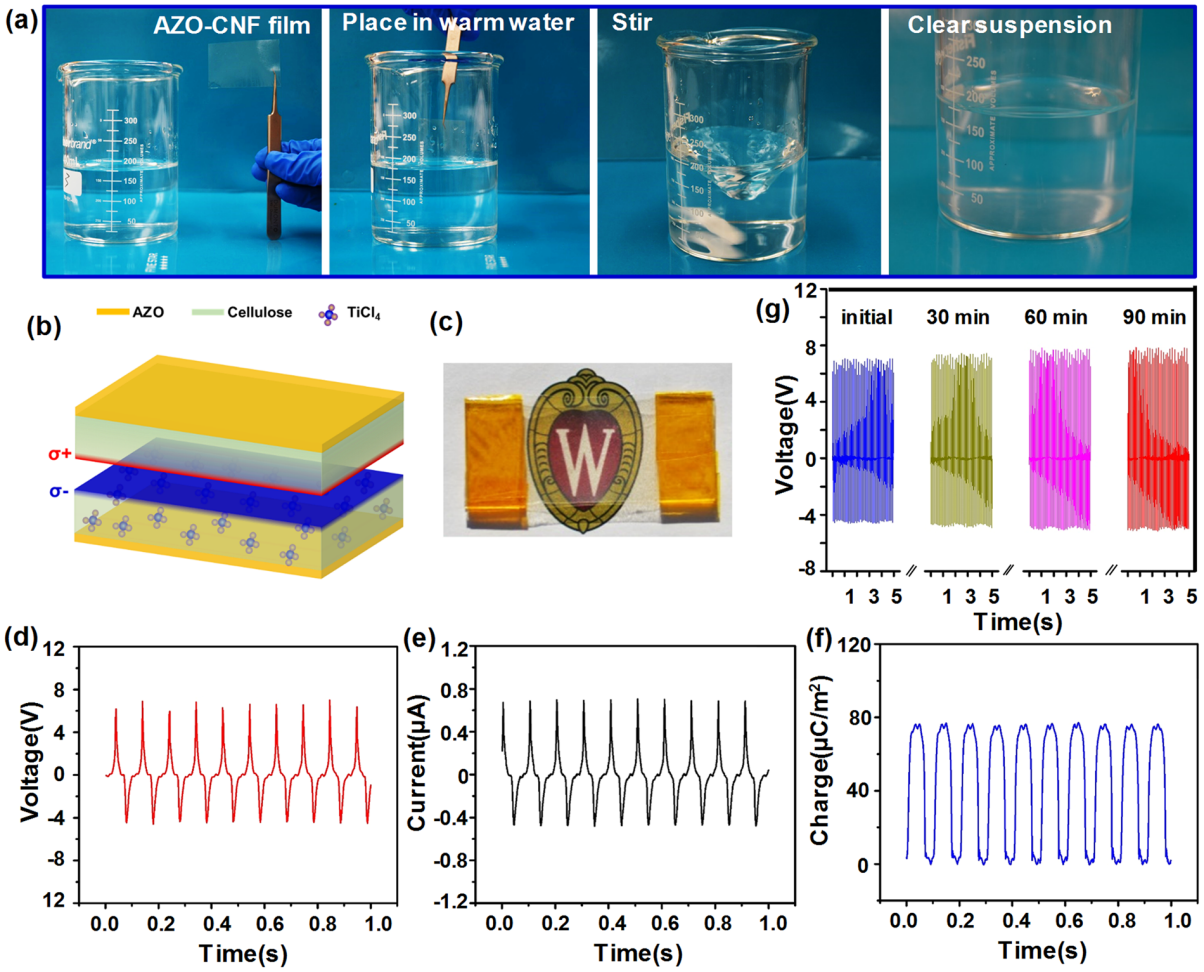
Degradation and TENG application of AZO-CNF papers. (**a**) A series of photos showing the process of dissolving an AZO-CNF paper in city water at 70 °C. The AZO-CNF paper was completed dissolved after 60-minute stirring. (**b**) Schematic image of a TENG made from a pair of AZO-CNF paper. (**c**) An optical image of the transparent all-CNF TENG device. (**d–f**) The voltage (**d**), current (**e**) and charge (**f**) outputs of the TENG device measured at 10 Hz frequency. (**g**) The long-term stability of AZO-CNF-based TENG. It showed a stable output after 90-minute operation.

One unique merit of AZO-CNF paper that distinguishes it from other synthetic flexible polymer substrates is its excellent degradability and recyclability. As shown in Fig. 4a, one piece of AZO-CNF paper was placed into a warm city water (70 °C). After 60-minute stirring, the AZO-CNF paper was completely dissolved into the water. The final solution was clear and no suspension can be observed. It became a CNF gel with a small amount of Zn and Al ions coming from the AZO coating. Using AZO as the TCO component ensures completely dissolving of all the film components. This solution can thus be further concentrated and filtered to produce CNF film again, suggesting an excellent recyclability.

Flexible transparent TCO substrates have broad application potentials in many modern electronic and optical systems. As an example of application, a pair of AZO-CNF paper was used to fabricate a new nature degradable TENG device for mechanical energy harvesting. The configuration of this TENG device is schematically shown in Fig. 4b. It was a simple vertical contact mode TENG where the AZO layer was used as the back electrodes and the CNF surface was used for triboelectric charge generation. Since the operation of TENG requires two contacting surfaces have drastically different electron affinities, one CNF paper was treated by oxygen plasma followed by TiCl_4_ infiltration. Since both oxygen and chlorine atoms have strong tendency to attract electrons, incorporation of these atoms could largely increase the electronegativity of the CNF paper. Due to the small quantity and discrete distribution of the infiltrated molecules, and the relatively small infiltration depth (a few microns) compared to the overall film thickness, this process usually imposes negligible influence to the internal resistance of the film^44–46^. X-ray photonic spectra (XPS) revealed significantly enhanced oxygen peaks and the appearance of strong Ti and Cl peaks (Fig. S5). Further XPS fine structure analysis confirmed the formation of more polar C-O bonds and successful incorporation of Cl and Ti elements into the cellulose network. These features made cellulose become strongly triboelectric negative. Since the untreated CNF is slightly triboelectric positive, a contact between them would induce significant charge transfer and thus achieve high triboelectric output.

Because both triboelectric materials and electrodes were made from AZO-CNF paper, this TENG device was flexible and transparent (Fig. 4c). Because no more metal contact was used in this TENG assembly, the entire device was also completely water degradable. The triboelectric output is shown in Fig. 4d–f, where open circuit voltage, short circuit current and corresponding charge transfer are all presented. The TENG was under a constant 10 Hz impact during the measurements. The maximum voltage and current was ~7 V and ~0.7 μA, respectively; and corresponding charge transfer was ~77 μC/m^2^. These outputs were comparable to plasma-treated CNF TENG and significantly higher than those from untreated AZO-CNF pairs (Fig. S6), suggesting the plasma and TiCl_4_ treatment were both effective in tuning the CNF’s electronegativity and enhancing the triboelectric output. Following the same principle being reported previously on AlO_x_ infiltration^44^, the TiCl_4_ infiltration approach was very effective and only a few cycles of exposure would bring the output enhancement to the saturation point. Figure S7 shows the voltage and power outputs of the TENG as a function of load resistances. As expected, the voltage output increased with the increase of the load resistance from 1 × 10^4^ to 5 × 10^10^. The maximum power of mW was obtained at 1 MΩ load resistance, comparable to other cellulose-based TENG devices^27^. The stability of the all-CNF TENG was tested for long operation time under continuous 2 Hz mechanical impacts. The voltage output was recorded every 30 minutes and no deterioration could be identified (Fig. 4g). Similar stability was also discovered from the plasma treated TENG samples (Fig. S7). Both measurements confirmed the excellent stability of the AZO coating on CNF even under continuous intense mechanical impacts.

## Discussion

In this work, an AZO thin film was deposited on a CNF paper by low-temperature ALD creating a new type of flexible transparent TCO substrate. Due to the relatively open structure of the CNF film, a fair amount of oxide was grown into the CNF film, which rooted the AZO thin film tightly on the CNF surface. As a result, even without a high-temperature treatment, the AZO coating exhibited a high tolerance to both tensile and compressive strains. Its *R*s remained at a low value in the range from 130 to 180 Ω · sq^−1^ across a broad strain range from 0.057% to 0.196%. By measuring *R*s and transparency of the AZO-CNF papers with a series of Al concentration, we found that the 3.76 at.% Al doped ZnO thin film exhibited the best combination of transparency and conductivity, which was at the same level of other common flexible transparent conductive substrates including ITO- and graphene-coated PET substrates. This data suggested that AZO-CNF paper could be used as a new type of flexible transparent electrode for many optoelectronic and electronic devices. In addition, the AZO-CNF paper also exhibited an excellent degradation behavior in warm water. This is the first report of flexible transparent conductive substrates that are also naturally degradable. It offers a unique solution for building green optoelectronic devices. As an intriguing application direction, flexible and transparent TENG devices were made merely from the AZO-CNF paper. Oxygen plasma and TiCl_4_ SIS approaches were both applied to tuning the electronegativity of the CNF film. A reasonably high TENG output was detected, placing this AZO-CNF device at the same level as other common synthetic polymer-based TENGs. In addition to TENG devices, the AZO-CNF paper may also find a broad application potential as the conductive substrates of many other flexible optoelectronic systems, such as solar cells, optical sensors, LEDs, particularly when the devices need to be naturally degradable.

## Methods

### Preparation of AZO-CNF papers

The as-processed CNF hydrogel^27^ was diluted with DI water and then filtered under ~0.55 MPa air pressure in a filtration system (Millipore Corporation, USA). A filter paper covered by a polytetrafluoroethylene membrane that have 0.1 um pore sizes

The AZO thin film was grown by ALD. The as-prepared CNF paper was placed on top of an alumina plate located in a home-built ALD reactor chamber. The ALD chamber was kept at 150 °C during the entire ALD process. The precursors used to grow AZO films were Dimethyl zinc (Zn(CH_3_)_2_) (DEZn), Trimethylaluminum (Al(CH_3_)_3_) (TMA) and water (H_2_O). Nitrogen (99.999%) was used as a purge and carrier gas with a flow rate of 40 sccm. One ZnO cycle consists of 0.5 s DEZn pulsing, 30 s N_2_ purging, 0.5 s H_2_O pulsing, and 30 s N_2_ purge. One Al_2_O_3_ cycle has 0. 5 s TMA pulsing, 30 s N_2_ purging, 0.5 s H_2_O pulsing and 30 s N_2_ purging. In the deposition process for synthesizing AZO coatings, deposition cycle ratio of the DEZn/TMA (X:Y) increased from 20:1 to 5:1 to achieve different Al doping concentrations. The un-doped ZnO coating was prepared by the same ALD process with only DEZn/H_2_O cycles. The total number of coatings for each sample was kept at 1500 cycles ZnO plus varied Al_2_O_3_ cycles (0, 75, 100, 150, 300) to finish all of the deposition cycles ratio (20:1, 15:1, 10:1, 5:1). The deposition time is 1525, 1601, 1626.7, 1677.5 and 1830 min, respectively.

### Property Characterization and Measurements

The surface and cross-sectional morphology and EDS analysis of AZO coatings were characterized by LEO-1530 scanning electron microscopy (SEM). The X-ray photoelectron spectroscopy (XPS, Thermo Fisher Scientific Inc., Waltham, MA) was applied to characterize the chemical compositions from the surface of the AZO-CNF paper. The survey binding energy range was set from 0 to 1200 eV. The fine structure of the N 1s, O 1s, Cl 2p and Ti 2p spectra were measured for characterizing the surface composition and chemical state. The crystal structure was conducted by X-Ray diffraction system (Thermo Fisher Scientific).

The absorption spectra were recorded using an Evolution 220 UV-vis spectrophotometer with integrated sphere (ISA 220) in the range from 200 nm to 800 nm. The sheet resistances of the films were measured using a homebuilt four-point probe system. The distance between electrodes was kept at 2 mm. Different bending curvatures were achieved by fixing the AZO-NCF paper on a series of cylindrical molds with radii of 43.5 mm, 35 mm, 25 mm, 16.5 mm, 12.8 mm, and 5.5 mm.

### Fabrication of TENG and the preparation of the materials

The CNF side of the AZO-CNF paper was treated by plasma and/or TiCl_4_ infiltration to tune the triboelectric polarity. The plasma modification was implemented in an Inductive Coupled Plasma chamber under 300 W O_2_ plasma radiation for 10 minutes. The TiCl_4_ infiltration was conducted in the ALD chamber by 10 cycles of 5-second TiCl_4_ vapor exposure followed by 60-second N_2_ purge. One processed AZO-CNF paper was paired with a pristine AZO-CNF paper to fabricate a vertical-contact TENG device. The size of the TENG was 60 mm × 20 mm. A 10 mm × 10 mm glass sheets were attached to the top film to achieve uniform contact within that area. The glass sheet was pressed down by a shaker with 17 N force at designed frequencies. The voltage output was measured using an Agilent DSO1012A oscilloscope, and the current and charge output was recorded by an Autolab PGSTAT302N station. The output power (P) as a function of load resistance was calculated following: P = V^2^/R, where *R* is the total resistance of the circuit and was determined from 1/R = 1/R_load_ + 1/R_Oscilloscope_.

## Electronic supplementary material

Supplementary materials
