# Supplementary material for: Nature Degradable, Flexible, and Transparent Conductive Substrates from Green and Earth-Abundant Materials
Source: Sci Rep. 2017 Jul 10;7:4936. doi: 10.1038/s41598-017-04969-y (PMC5503997; doi:10.1038/s41598-017-04969-y)
Supplement: Supplementary file 1 — Supplementary materials [file 41598_2017_4969_MOESM1_ESM.pdf]

Supplementary Materials

Nature Degradable Flexible and Transparent Conductive Substrates from Green and Earth-Abundant Materials

Bing Yang[1,2], Chunhua Yao[1], Yanhao Yu[1], Zhaodong Li[1], Xudong Wang[1,*]

1. Department of Materials Science and Engineering, University of Wisconsin-Madison, Madison, WI 53706, USA

2. School of Power and Mechanical Engineering, Wuhan University, 430072 Wuhan, China

Email: xudong.wang@wisc.edu

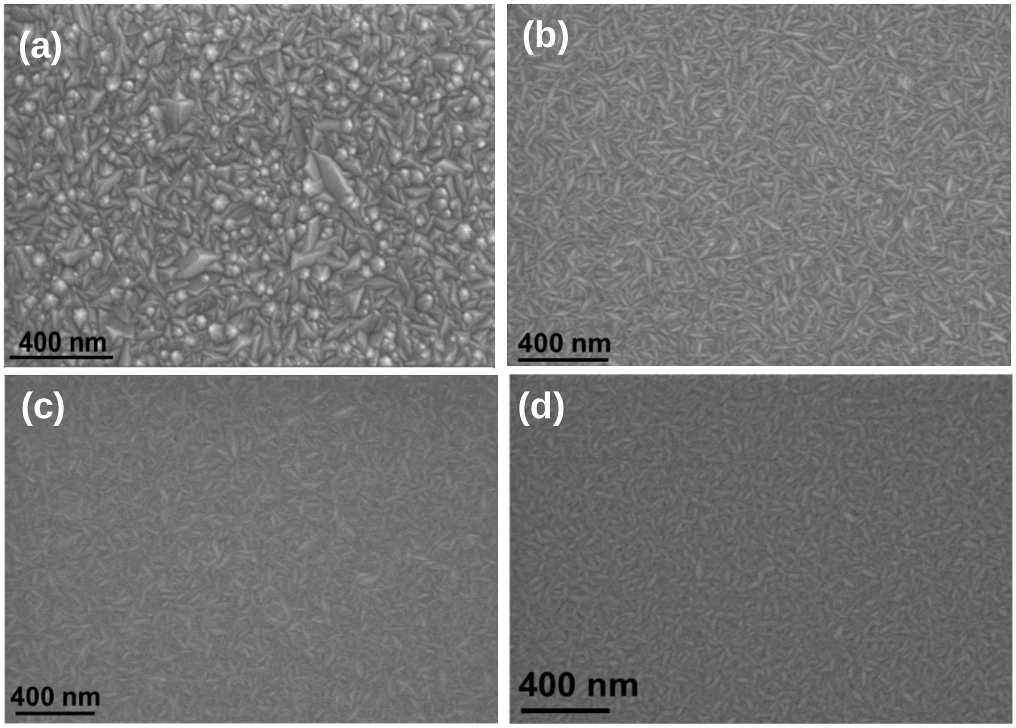

Figure S1. Surface morphologies of AZO-coated CNF paper with different Al concentrations. **(a)** ZnO. **(b)** AZO with 2.24 at.% Al. **(c)** AZO with 5.1 at.% Al. **(d)** AZO with 10.91 at.% Al.

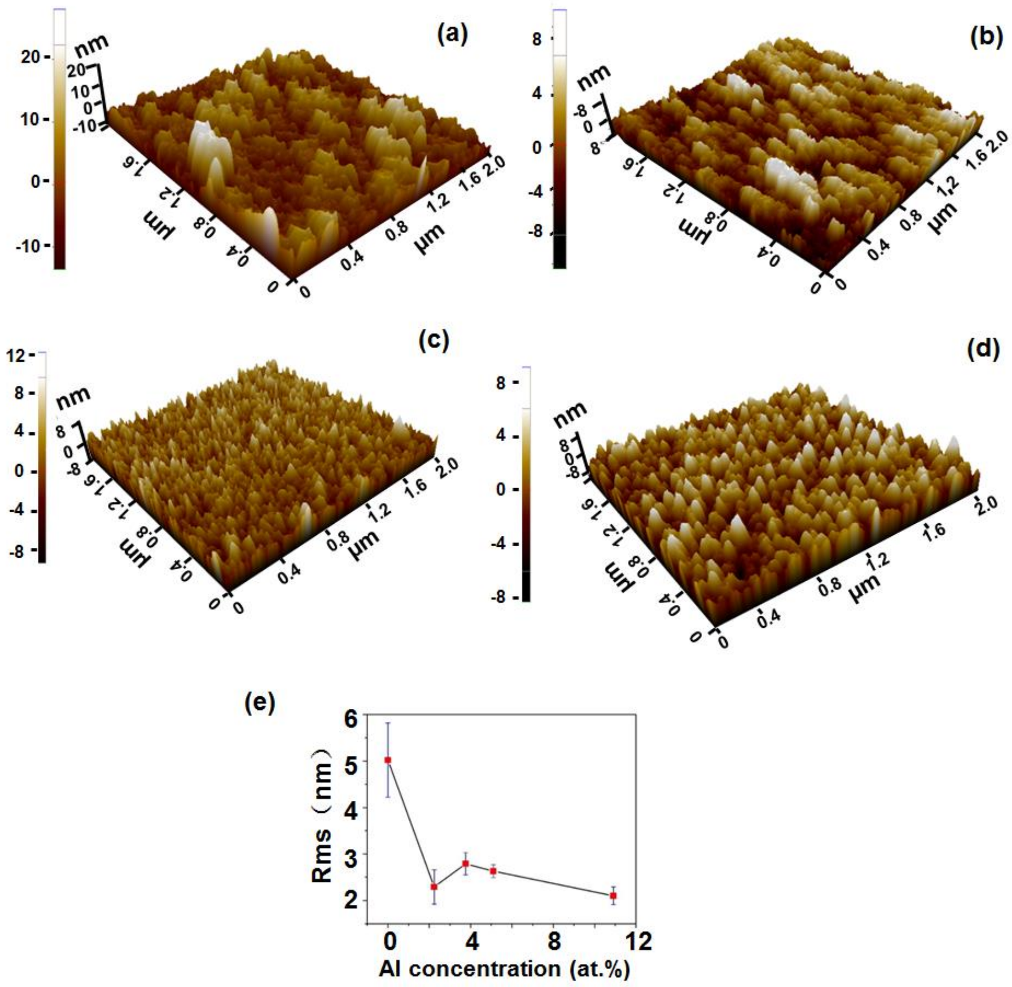

Figure S2. AFM topographic images of AZO surface with different Al concentration: **(a)** ZnO. **(b)** AZO with 2.24 at.% Al. **(c)** AZO with 5.1 at.% Al. **(d)** AZO with 10.91 at.% Al. **(e)** The surface roughness as a function of Al concentration. It decreased gradually as the Al concentration increased.

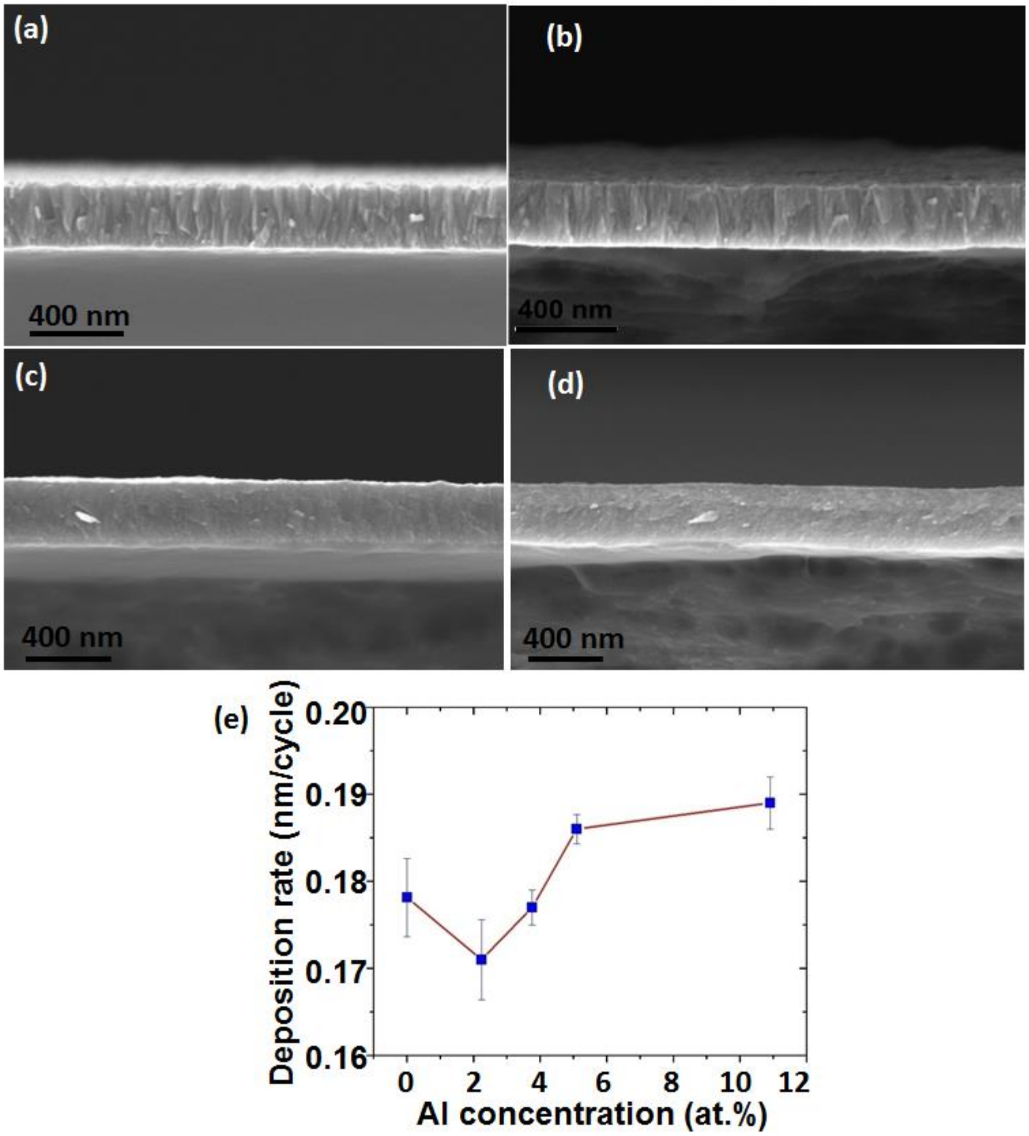

Figure S3. Cross-sectional images and deposition rate of AZO-CNF paper with different Al concentrations: **(a)** ZnO. **(b)** AZO with 2.24 at.% Al. **(c)** AZO with 5.1 at.% Al. **(d)** AZO with 10.91 at.% Al. **(e)** AZO deposition rate as a function of Al concentration.

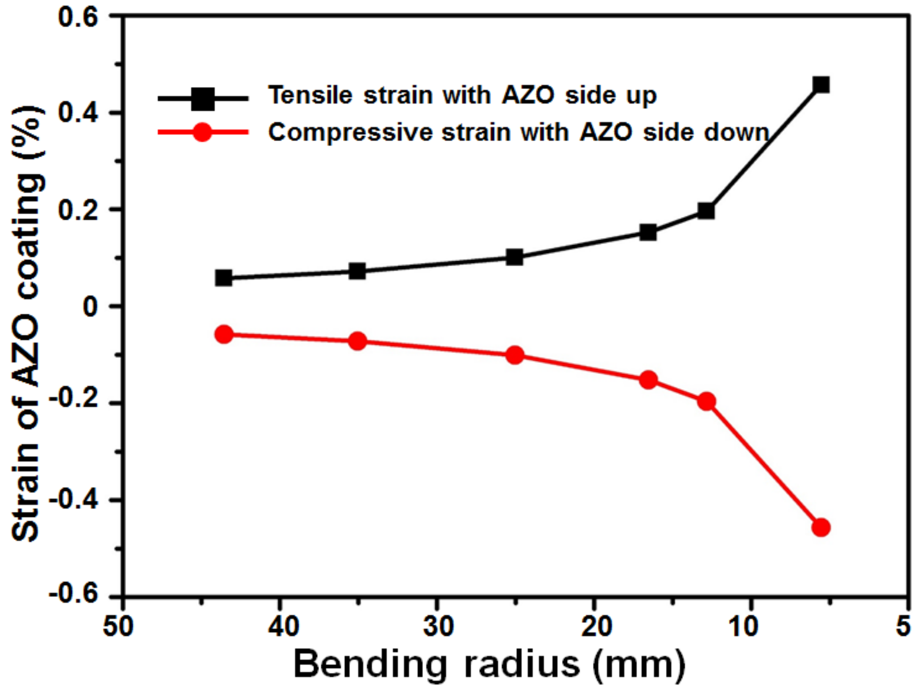

Figure S4. Strain subjected by the AZO film as the AZO-CNF paper bended at different bending radius. The black squares represent the tensile strains when the AZO side was facing up. The red spheres represent the compressive strains when the AZO side was facing down.

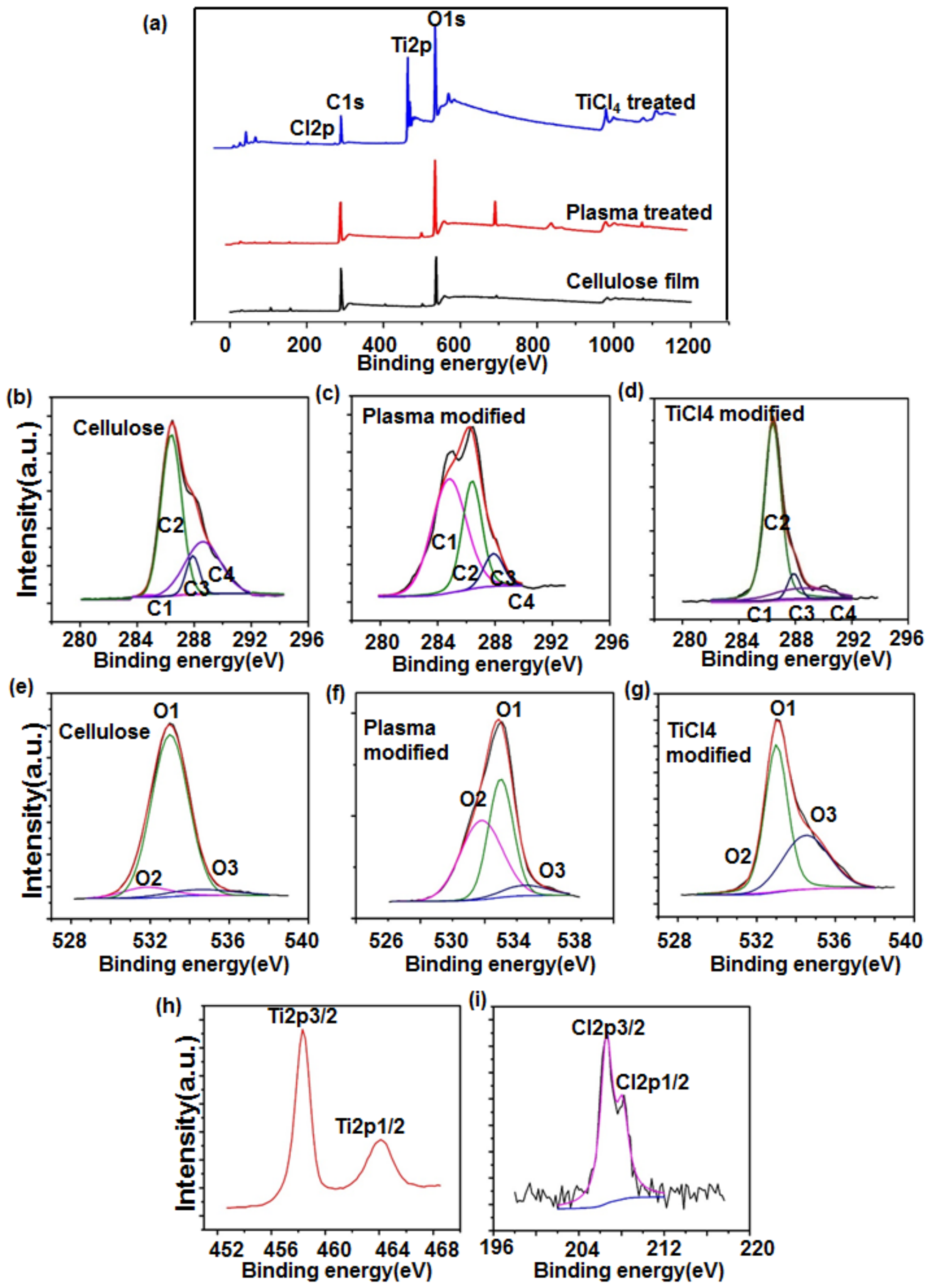

Figure S5. (a) The full span XPS survey of CNF films before and after oxygen plasma and $TiCl_4$ treatments. **(b-d)** Deconvoluted spectra for the C 1s peak of pristine CNF film **(b)**, Plasma treated CNF film **(c)**, and $TiCl_4$ treated CNF film **(d)**. **(e-g)** Deconvoluted spectra the O 1s peak of pristine CNF film **(e)**, Plasma treated CNF film **(f)**, and $TiCl_4$ treated CNF film **(g)**. **(h, i)** Deconvoluted spectra for the Ti 2p peak **(h)** and Cl 2p **(i)** of the $TiCl_4$ treated CNF film.

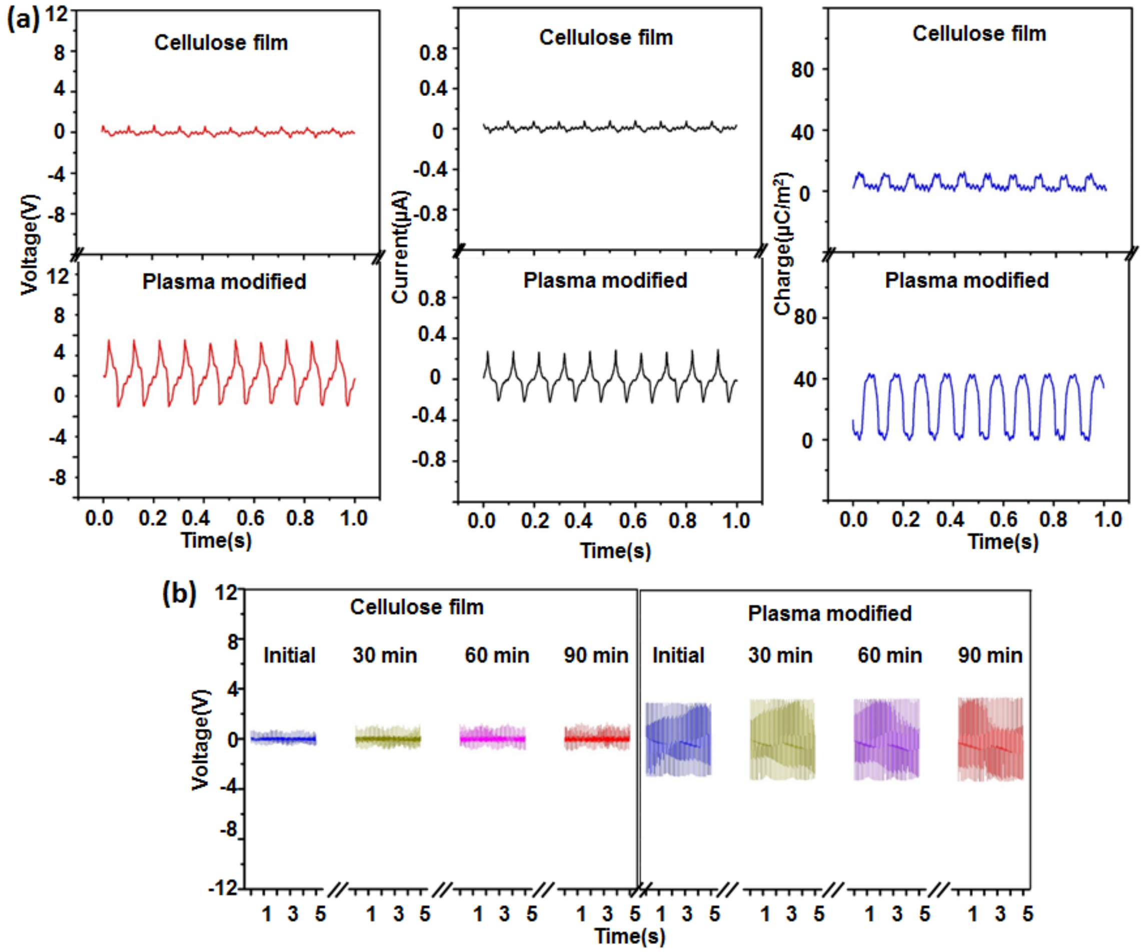

Figure S6. (a) The voltage, current and charge outputs of TENGs made from a pair of pristine AZO-CNF papers (top) and a pair of AZO-CNF papers with one treated by plasma (bottom). The vibration frequency was kept at 10Hz. **(b)** Long-term stability tests of the AZO-CNF paper-based TENGs. Left: both were pristine; Right: one paper was treated by plasma.

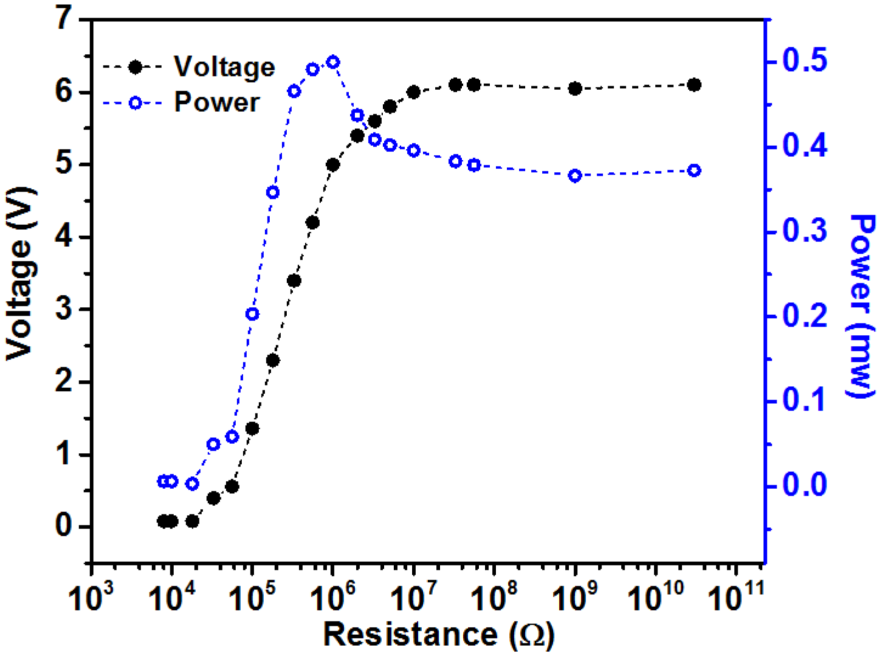

Figure S7. The voltage and power outputs of AZO-CNF TENG device as a function of the load resistance.
